# *Yersinia pestis* Lineages in Mongolia

**DOI:** 10.1371/journal.pone.0030624

**Published:** 2012-02-17

**Authors:** Julia M. Riehm, Gilles Vergnaud, Daniel Kiefer, Tserennorov Damdindorj, Otgonbaatar Dashdavaa, Tungalag Khurelsukh, Lothar Zöller, Roman Wölfel, Philippe Le Flèche, Holger C. Scholz

**Affiliations:** 1 Bundeswehr Institute of Microbiology, Munich, Germany; 2 Université of Paris-Sud, Institut de Génétique et Microbiologie, Orsay, France; 3 Centre National de la Recherche Scientifique (CNRS), Orsay, France; 4 DGA/MRIS-Mission pour la Recherche et l'Innovation Scientifique, Bagneux, France; 5 National Center of Infectious Diseases with Natural Foci, Ulaanbaatar, Mongolia; 6 Division of Analytical Microbiology, Direction générale de L'armement (DGA)/Maîtrise NRBC, Vert le Petit, France; East Carolina University School of Medicine, United States of America

## Abstract

**Background:**

Whole genome sequencing allowed the development of a number of high resolution sequence based typing tools for *Yersinia (Y.) pestis*. The application of these methods on isolates from most known foci worldwide and in particular from China and the Former Soviet Union has dramatically improved our understanding of the population structure of this species. In the current view, *Y. pestis* including the non or moderate human pathogen *Y. pestis* subspecies *microtus* emerged from *Yersinia pseudotuberculosis* about 2,600 to 28,600 years ago in central Asia. The majority of central Asia natural foci have been investigated. However these investigations included only few strains from Mongolia.

**Methodology/Principal Findings:**

Clustered Regularly Interspaced Short Prokaryotic Repeats (CRISPR) analysis and Multiple-locus variable number of tandem repeats (VNTR) analysis (MLVA) with 25 loci was performed on 100 *Y. pestis* strains, isolated from 37 sampling areas in Mongolia. The resulting data were compared with previously published data from more than 500 plague strains, 130 of which had also been previously genotyped by single nucleotide polymorphism (SNP) analysis. The comparison revealed six main clusters including the three *microtus* biovars Ulegeica, Altaica, and Xilingolensis. The largest cluster comprises 78 isolates, with unique and new genotypes seen so far in Mongolia only. Typing of selected isolates by key SNPs was used to robustly assign the corresponding clusters to previously defined SNP branches.

**Conclusions/Significance:**

We show that Mongolia hosts the most recent *microtus* clade (Ulegeica). Interestingly no representatives of the ancestral *Y. pestis* subspecies *pestis* nodes previously identified in North-western China were identified in this study. This observation suggests that the subsequent evolution steps within *Y. pestis pestis* did not occur in Mongolia. Rather, Mongolia was most likely re-colonized by more recent clades coming back from China contemporary of the black death pandemic, or more recently in the past 600 years.

## Introduction

*Yersinia (Y.) pestis* subspecies *pestis* is the causative agent of human plague. Cases are annually registered by the WHO and nowadays mostly occur in Asia, Africa, and America [1]. Three major pandemics affecting geographic regions previously devoid of established foci are known to Western history, and *Y. pestis* spread to all continents except Australia and Antarctica [2], [3]. The zoonotic plague disease can be transmitted from natural host reservoirs, mostly rodents, via various vectors to other mammals including humans. It is therefore a multi-host and multi-vector pathogen [4].

In the past ten years the use of modern molecular genetics to investigate isolates recovered from most natural foci as well as remains from victims of past pandemics has dramatically increased our understanding of the population structure, origin and spread of this major pathogen. The current view is that *Y. pestis* can be divided in biovars or ecotypes, grouped into subspecies *pestis* and subspecies *microtus*. Subspecies *microtus* comprises a number of biovars mostly harmless for humans. *Microtus* was initially investigated by microbiologists from the Former Soviet Union (FSU) under the name pestoides and subsequently under the different phenotype-based biovar designations Caucasica, Ulegeica, Altaica, Hissarica, and Talassica [4]. More recently two additional biovar designations were defined to cover Chinese *microtus* lineages, namely Xilingolensis and Qinghaiensis [5]. Whole genome sequencing and large scale SNP analysis has provided a robust branching order of the main clades within *Y. pestis*. The Caucasica biovar recovered so far only from nearby foci in Georgia, Armenia, Azerbaijan and Russia, corresponds to branch 0.PE2 in the SNP typing nomenclature proposed by Achtman and colleagues [4], [6], [7]. Together with 0.PE7, 0.PE2 branched out most ancestrally from the linear tree leading from *Y. pseudotuberculosis* to *Y. pestis* subspecies *pestis* biovar Orientalis. The strains defining the 0.PE7 clade were first identified as peculiar by Li et al. [5]. Only two strains corresponding to this clade have been reported so far (Figure 2 in [5]). C1961001 and C1962002 were recovered from Xinghai district in Qinghai province and importantly C1962002 was isolated from a human patient according to published information [5]. This would qualify clade 0.PE7 as a subspecies *pestis* biovar rather than *microtus*. The next branch 0.PE3 is represented by the unique Angola *microtus* strain, the geographic origin of which is uncertain [6]. It is followed by the branch leading to both 0.PE4 (*microtus* biovars Xilingolensis and Qinghaiensis) and 0.PE1 (represented by pestoides A, B, C, D with no correspondence provided in terms of *microtus* biovar designation [6], [7]). The rest of the ancestral 0 branch is currently populated predominantly by strains originating from China focus B in the Xinjiang province which define three nodes 0.ANT1, 0.ANT2, and 0.ANT3 [6], [7] potentially pathogenic for humans. The investigation of human remains associated with the Black Death demonstrated that the associated *Y. pestis* strains were almost coincident with the 3.ANT node [8]–[10] indicating that branches 1 (Orientalis biovar and Antiqua strains from Africa) and 2 (Antiqua strains from Tibet, Manchuria and Medievalis biovar) are less than 700 years old. The finding of many 0.ANT branches in China suggests that the Black death *Y. pestis* evolved in or near western China, and spread via a number of radiations to Southeast Asia, Africa, Europe, South and North America, leading to country-specific lineages [5], [6].

Up to now, several hundred *Y. pestis* strains from the majority of known foci all over the world were analyzed and typed using MLVA based on VNTR loci selected from a collection of more than 60 loci shown to be polymorphic within *Y. pestis* [3], [5], [7], [11]–[17]. A significant fraction of these strains has also been typed by Clustered Regularly Interspaced Short Prokaryotic Repeats (CRISPR) [18] analysis and large-scale SNP typing [6]. Regarding *Y. pestis*, the comparability of those methods particularly MLVA and SNPs, and hence the applicability of *progressive hierarchical resolving assays using nucleic acids* (PHRANA) as earlier described for *Bacillus anthracis* has not been investigated so far [19], [20].

Numerous Chinese and FSU isolates were amongst the investigated strains, but only four Mongolian isolates from two foci have been analyzed. Mongolia is a place of numerous highly active plague foci [4], [21]. *Y. pestis* can be isolated in almost any province of Mongolia, and human plague is recorded since 1897 there, but was present for a much longer time in Siberian marmots [22]. In particular, Western Mongolia is an exceptional region in terms of *Y. pestis* diversity, as the Altaica and Ulegeica *microtus* biovars as well as *Y. pestis* subspecies *pestis* coexist in a relatively limited geographic space [4]. The present work was carried out to characterize 100 Mongolian *Y. pestis* strains from 37 different natural sampling places applying recent molecular analysis tools. CRISPR analysis and MLVA with 25 loci were used as a quick first line classification assay. Resulting data were compared to strains previously characterized by CRISPR, MLVA and SNP analysis [5], [6], [18]. MLVA clusters containing no strain previously typed by SNP analysis were assigned to SNP nodes by typing key SNPs on selected strains.

## Results and Discussion

### Direct comparison and aggregation of published MLVA and SNPs clustering

**Figure 1:**
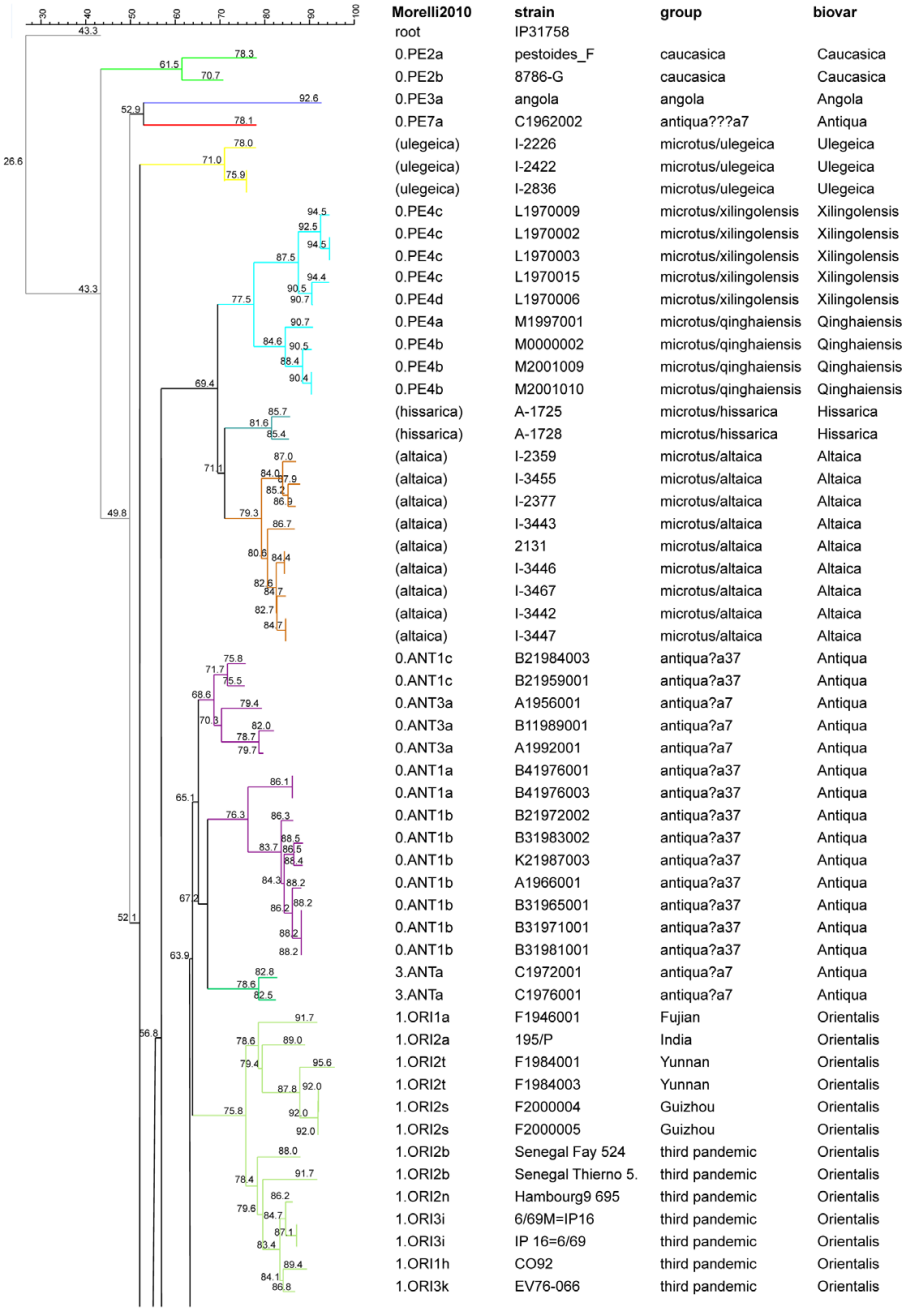
MLVA clustering and SNP branch assignment of 66 previously published *Y. pestis microtus* and *pestis* 0, 1 and 3 branches. *Microtus* and strains from the 0 and 1 branches so far investigated by MLVA25 and by SNP analysis are shown [5], [6]. Three Ulegeica, two Hissarica and nine Altaica strains not investigated by SNP analysis are also included. For completion, Table 1 gives further information about assignment of biovar, genotype, and origin. Colors reflect MLVA clustering as suggested by Li et al. [5]. The SNP branch assignment of each strain as defined by Morelli et al. is indicated (column *Morelli2010*) together with the strain ID and biovar designation [6]. The results of CRISPR analysis according to Cui et al. are shown in column *group* [18]. Bootstrap support values are indicated. The figure shows the satisfying terminal branches clustering achieved by MLVA but the sometimes incorrect and usually low bootstrap values of deep branching nodes illustrating the complementarity of the two methods.

**Figure 2:**
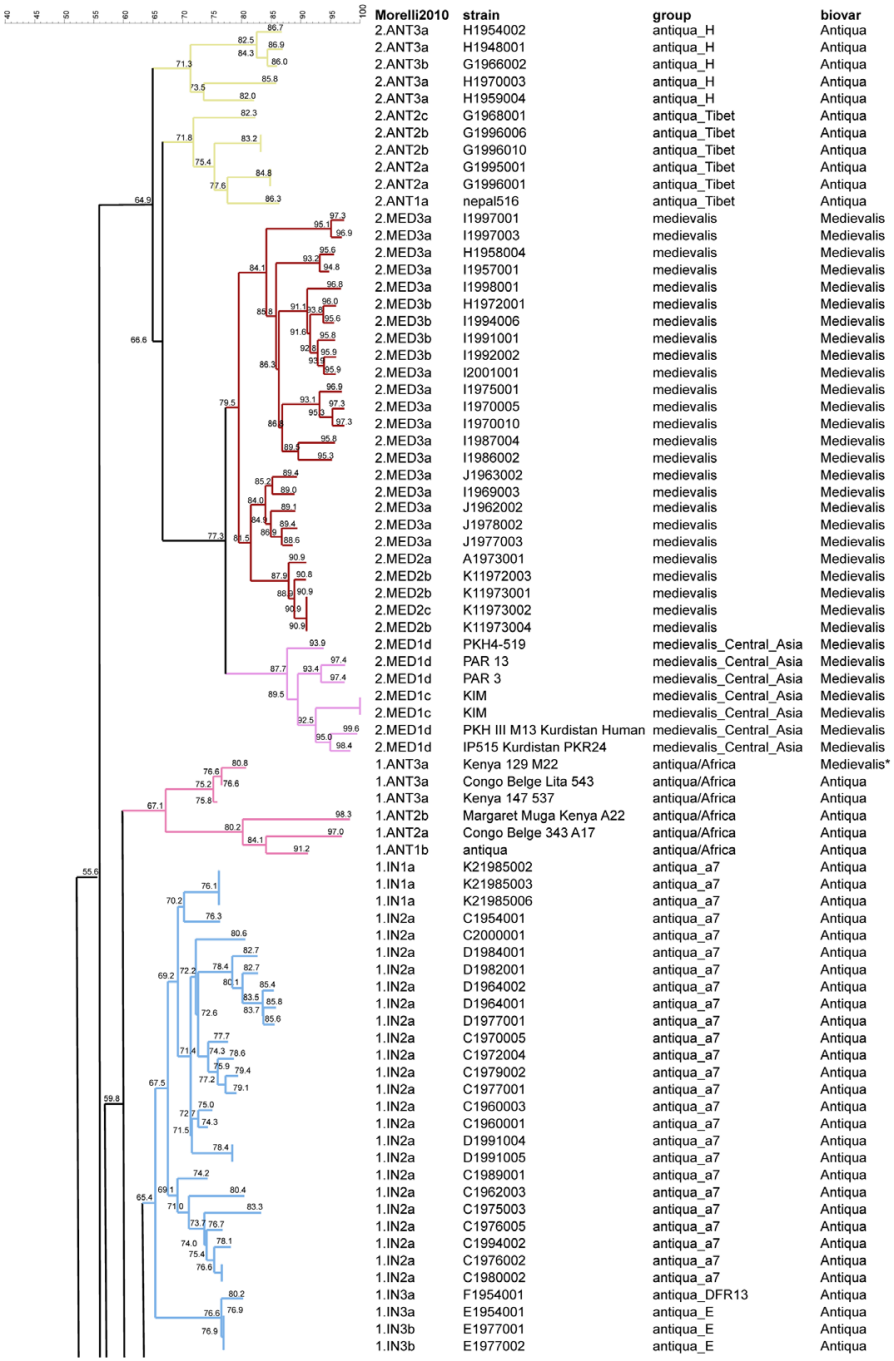
MLVA clustering and SNP branch assignment of 68 previously published *Y. pestis pestis* branches 1 and 2. Sixty-eight strains from the 1 and 2 branches previously investigated by both MLVA25 and SNP analysis are displayed [5], [6]. For completion, Table 1 gives further information about assignment of biovar, genotype, and origin. Colors reflect MLVA clustering as suggested by Li et al. [5]. The SNP branch assignment of each strain as defined by Morelli et al. is indicated (column *Morelli2010*) together with the strain ID and biovar designation [6]. Bootstrap support values are indicated for each node. The results of CRISPR analysis according to Cui et al. are given in column *group* [18]. * This strain shows a Medievalis phenotype due to a different mutation in the napA gene compared to the mutation causing the Medievalis phenotype in the Medievalis biovar, as demonstrated by Pourcel et al. [13].

**Figure 3:**
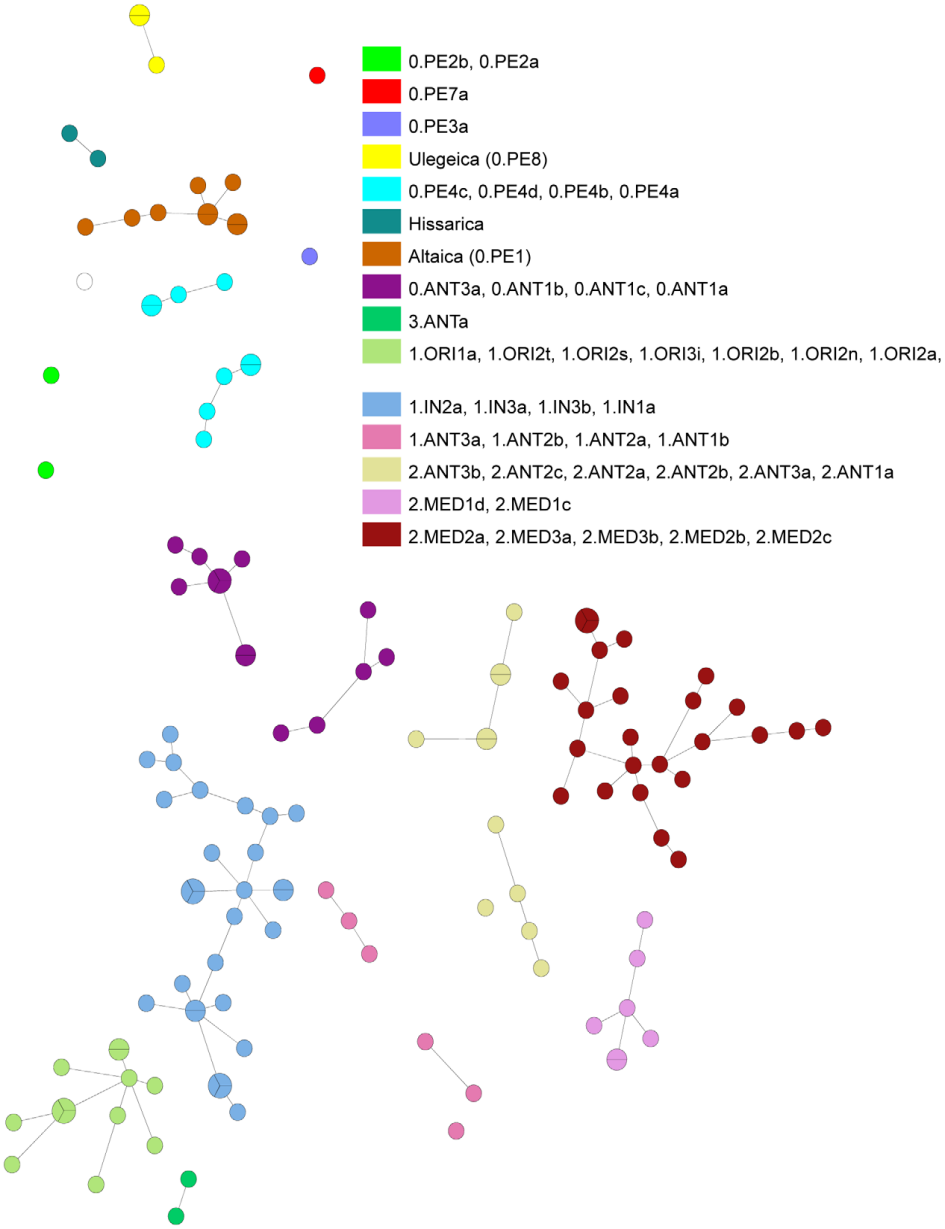
Minimal spanning tree of the strains as shown in **Figures 1** and **2** using the same color code. The figure is based on the same data set as Fig. 1 and 2. Table 1 gives further information about assignment of biovar, genotype, and origin. Basic correlation and grouping of genotypes is similar compared to previously published Fig. 2 in Morelli et al. [6].

The work by Morelli et al. [6] was used to evaluate the relevance of CRISPR and MLVA cluster analysis carried out by Li et al., and Cui et al. [5], [18], and to link the different clusters. One hundred and thirty-one strains (subsequently called linking strains) were investigated by both Li et al. and Morelli et al. [5], [6]. MLVA clustering of this common set of strains using data from Li et al. [5] is shown in Figures 1 and 2. For each strain the SNP branch determined by Morelli et al. [6] is indicated. For instance, the 0.PE4a and 0.PE4b branches correspond to the Qinghaiensis *microtus* biovar, whereas the 0.PE4c and 0.PE4d branches correspond to the Xilingolensis *microtus* biovar (Figure 1 and 3, Table 1). The correspondence between 0.PE1 strains (pestoides A, B, C, D) and Altaica could be deduced by comparing MLVA data from Achtman et al. [7] and Li et al. [5], taking advantage of loci included in both assays (Figure 1, Table 1). Figures 1 and 3 also include Ulegeica (from Mongolia) and Hissarica (from Uzbekistan) strains investigated only by CRISPR analysis and MLVA [5], [18]. MLVA clustering suggests with moderate support that the Hissarica biovar is closest to the 0.PE1 and 0.PE4 *microtus* branches, but SNP typing will be required to confirm this assumption given the long MLVA branch leading to the Hissarica strains (Figure 1). The interest of combining the MLVA discriminatory power, clustering efficiency and low cost with the phylogenetic robustness of SNPs illustrated here is in agreement with similar findings obtained for *Bacillus anthracis* [19], [20]. Also, a recent investigation of 262 *Y. pestis* strains collected in Madagascar confirmed the interest of combining MLVA and SNP typing assays [23].

**Table 1:** Overview of *Y. pestis* subspecies, biovar, genotype, and natural foci as suggested by different authors [4], [5], [6], [7], [18], and as deduced in this study.

Strain (example)	Subspecies	Biovar	Genotypes*determined by SNP analyses	Appearance/natural plague foci[5], [6]
C1962002			0.PE7	China/C
Pestoides F	*microtus*	Caucasica	0.PE2	Armenia/#4,#5,#6
Angola	*microtus*	(Pestoides)	0.PE3	Origin uncertain
Pestoides A, B, C, D	*microtus*	Altaica (deduced/this study)	0.PE1	Kazakhstan, Mongolia/#36, 7, 8a
M1997001	*microtus*	Qinghaiensis	0.PE4a	China/M
M2001009	*microtus*	Qinghaiensis	0.PE4b	China/M
L1970003	*microtus*	Xilingolensis	0.PE4c	China, Mongolia/L, 23,33
L1970006	*microtus*	Xilingolensis	0.PE4d	China/L
**MNG 2972**	*microtus*	Ulegeica	**0.PE8 - this study**	Mongolia/BP, 8, 10, 15
A-1725	*microtus*	Hissarica	0.PE9 (suggested)	Tajikistan, Uzbekistan/#34
B41976001	*pestis*	Intermedium	0.ANT1	China/A, B, K2
A1956001	*pestis*	Intermedium	0.ANT3	China, Kyrgyzstan/A,B,#33
C1972001	*pestis*	Antiqua	3.ANT	China, Russia, Mongolia/B, C, #37, 1, 3–6, 8a, 9–14, 16–22, 24–32, 34, 35
Antiqua	*pestis*	Antiqua	1.ANT	Africa
K21985002	*pestis*	Antiqua	1.IN1	China/C, K2
C1954001	*pestis*	Antiqua	1.IN2	China/C, D, F, H
E1979001	*pestis*	Antiqua	1.IN3	China/E,F
CA88-4125	*pestis*	Orientalis	1.ORI1	USA
F1991016	*pestis*	Orientalis	1.ORI2	China
IP674	*pestis*	Orientalis	1.ORI3	Turkey
Nepal516	*pestis*	Antiqua	2.ANT1	Nepal
G1995001	*pestis*	Antiqua	2.ANT2	China/C, G
H1948001	*pestis*	Antiqua	2.ANT3	China, Russia, Mongolia/B, G, H, #38, KP, 2, 34
KIM	*pestis*	Medievalis	2.MED1	Russia, Kurdistan, Kazakhstan, China/#16,#18,#21,#27,#43, O
K1973002	*pestis*	Medievalis	2.MED2	China/A,K1,K2
H1958004	*pestis*	Medievalis	2.MED3	China/D,G,H,I,J,L

*abbreviations as defined by Achtman et al. [7] and Morelli et al. [6]: PE – pestoides (*microtus*), ANT – Antiqua, IN – Intermedium, ORI – Orientalis, and MED – Medievalis; Intermedium in Morelli et al. [6] has not the same meaning as intermedium defined by Li et al. [5] which refers to Rhamnose positive *Y. pestis pestis* isolates.

# prefix refers to foci as described by Anisimov et al. [4]. Numbers without # refer to Mongolian foci as shown in Figure 4.

### MLVA clustering of the Mongolian isolates and tentative assignment of MLVA clusters to SNP branches

One hundred Mongolian *Y. pestis* strains were analyzed by CRISPR and MLVA analysis and compared to previously published data of 366 [18], and more than 500 strains [5], respectively.

**Figure 4:**
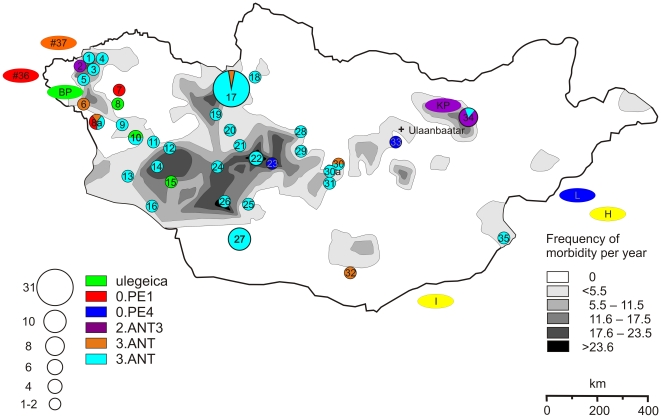
Sampling sites in Mongolia and observed genotypes. The map of Mongolia shows the sampling sites 1 to 35 and associated clusters (in color). Sizes of circles correlate to the number of collected strains. The exact geographic position of plague foci, and further background data of strains is listed in Table S1. Genotypes are explained in Table 1. Some previously published natural plague foci are shown in ovals. Colors match the corresponding lineage found in Mongolia: Mountain-Altai, Russia (#36), a reservoir for 0.PE1/Altaica strains. Tuva (Mongun-Taigin), Russia (#37), populated with 3.ANT genotype strains. Khentii province (KP), Mongolia [4], associated with the 2.ANT3 lineage. Bayanölgie province (BP), Mongolia [18] associated with the Ulegeica biovar. Chinese natural plague foci are present in Ningxia, Hebei, Shanxi, and Inner Mongolia, (I), Inner Mongolia, Jilin, Heilongjiang (H), and Inner Mongolia (L). Different lineages have been isolated here as shown by Li et al. [5].

**Figure 5:**
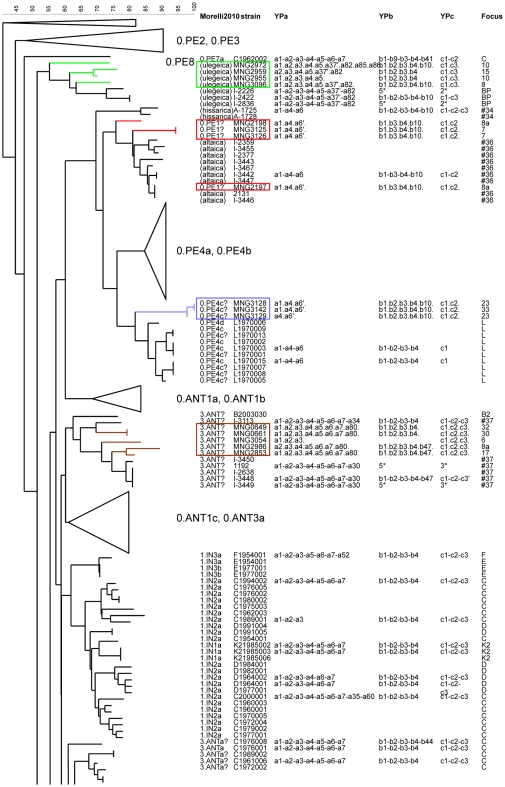
MLVA25 assignment of four clusters of the investigated Mongolian *Y. pestis* strains. MLVA25 tree of 16 investigated Mongolian *Y. pestis* strains (marked with color and boxes) representing four of the 6 clusters, and various *Y. pestis* strains originating from *microtus* and *pestis* biovars. For each strain, the tentative SNP branch or node according to Morelli et al. [6] as deduced by the presence of a linking strain in the same MLVA cluster is indicated by a question mark. Strain name, CRISPR profile as investigated in this study, and the sampling site (Focus) are listed.

**Figure 6:**
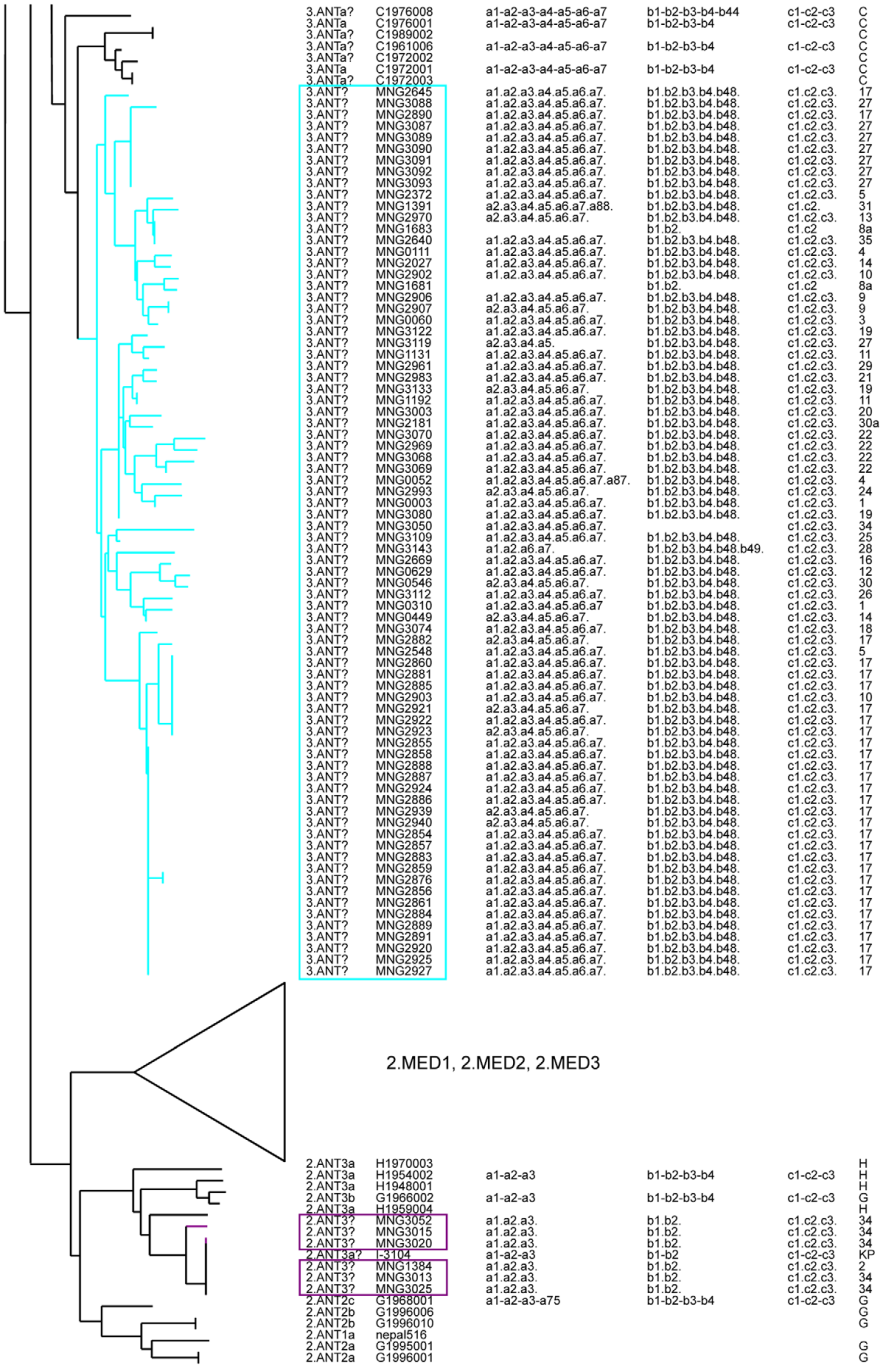
MLVA25 assignment of two clusters of the Mongolian *Y. pestis* strains. MLVA25 tree of two clusters comprising 84 investigated Mongolian *Y. pestis* strains (marked with color and boxes) compared to various previously typed *Y. pestis* strains. For each strain, the tentative SNP branch or node according to Morelli et al. [6] as deduced by the presence of a linking strain in the same MLVA cluster is indicated by a question mark. Strain name, CRISPR profile as investigated in this study, and the sampling site (Focus) are listed.

The 25 VNTR loci could be amplified in 96 of the 100 isolates. Sixty-five different MLVA25 genotypes are identified. Fifty-four of these are new compared to the current MLVA25 data [5]. The 100 isolates fall within six main clusters. Three clusters are *Y. pestis* subspecies *microtus* (11 isolates), the three others are *Y. pestis* subspecies *pestis* (89 isolates). The 11 *microtus* subspecies isolates fall into either Altaica (4 isolates from foci 7 and 8a), Xilingolensis (3 isolates from foci 23 and 33), or Ulegeica (4 isolates from foci 8, 10, 15) (Figure 4). The remaining 89 strains belong to the biovar Antiqua, and are distributed to all known Mongolian foci (Figure 4). Figures 5 and 6 show the resulting assignment for the 100 isolates together with previously investigated isolates from Li et al. [5].

Three of these clusters can be confidently assigned to a SNP branch owing to the co-clustering of at least one linking strain in the cluster: 0.PE1 (Altaica), 0.PE4 (Qinghaiensis/Xilingolensis), 2.ANT3 (Figure 5 and 6, Table 2). These three clusters comprise 4, 3, 6 isolates corresponding to 3, 2, and 2 MLVA25 genotypes respectively. The four 0.PE1 (Altaica) isolates are very closely related to previously investigated strains from Mountain-Altai focus #36 in Anisimov et al. [4] (Figures 1, 4 and 5). One isolate from focus 8a shows the same MLVA25 genotype as two previously reported strains #2131 and I-3446 in Li et al. [5]. The three 0.PE4 (Qinghaiensis/Xilingolensis) isolates are closely related to previously published Xilingolensis (0.PE4c/d) strains isolated from the L focus in China (Figures 1, 4, and 5). The six Mongolian *Y. pestis pestis* Antiqua isolates show MLVA25 genotypes typical of the Antiqua H MLVA cluster defined by Li et al. [5] and the 2.ANT3a SNP branch defined by Morelli et al. [6] (Figures 4 and 6/purple, Table 1). The MLVA25 genotype is identical or almost identical to one strain from the same focus called KP in Li et al. [5]. The H focus as defined by Zhou et al. [24], and Li et al. [25] is located in Manchuria, China, south of the L focus which hosts the Xilingolensis *microtus* biovar (Figure 4).

**Table 2:** Selected SNPs were determined for the Mongolian *Y. pestis* strains according to the previously published Fig. 2 in Morelli et al. [6].

		Branches and selected SNPs																		
Strains	Genotype	III-0.PE3.a		III-0.PE4.a		VI–III		VII–VI		0.ANT3.a-VII		3.ANT.a-0.ANT3.a		XI-3.ANT.a	XIII–XI	1.IN2.a-XIII	XI–XII	3.ANT.a-VIII	VIII-2.ANT3.a	2.ANT3.a-2.ANT2.a
		s914	s1278	s778	s100	s71	s78	s79	s86	s80	s84	s212	s545	s12	s3	s183	s271	s15	s231	s507
**CO92**		A	C	**G**	**G**	T	C	T	T	G	T	C	A	T	T	C	**C**	**C**	**C**	**A**
**ancestral genotype**		**G**	**T**	**G**	**G**	**C**	**T**	**C**	**C**	**A**	**C**	**A**	**G**	**C**	**C**	**G**	**C**	**C**	**C**	**A**
**derived genotype**		A	C	A	A	T	C	T	T	G	T	C	A	T	T	C	T	T	T	G
MNG 3128	0.PE4	A	C	A	A	**C**	**T**	**C**	**C**											
MNG 3129	0.PE4	A	C	A	A	**C**	**T**	**C**	**C**											
MNG 2197	0.PE1	A	C	A	**G**	**C**	**T**	**C**	**C**											
MNG 2198	0.PE1	A	C	A	**G**	**C**	**T**	**C**	**C**											
MNG 3126	0.PE1	A	C	A	**G**	**C**	**T**	**C**	**C**											
MNG 2959	*Ulegeica*	A	C	**G**	**G**	**C**	**T**	**C**	**C**											
MNG 2955	*Ulegeica*	A	C	**G**	**G**	**C**	**T**	**C**	**C**											
**MNG 2972**	**0.PE8** *(Ulegeica)*	A	C	**G**	**G**	**C**	**T**	**C**	**C**	**A**	**C**	**A**	**G**	**C**	**C**	**G**	**C**	**C**	**C**	**A**
MNG 649	3.ANT?					T	C	T	T	G	T	C	A	**C**	**C**	**G**	**C**	**C**	**C**	**A**
MNG 3054	3.ANT?					T	C	T	T	G	T	C	A	**C**	**C**	**G**	**C**	**C**	**C**	**A**
MNG 2986	3.ANT?					T	C	T	T	G	T	C	A	**C**	**C**	**G**	**C**	**C**	**C**	**A**
MNG 2853	3.ANT?					T	C	T	T	G	T	C	A	**C**	**C**	**G**	**C**	**C**	**C**	**A**
MNG 2645	3.ANT?					T	C	T	T	G	T	C	A	**C**	**C**	**G**	**C**	**C**	**C**	**A**
MNG 3088	3.ANT?					T	C	T	T	G	T	C	A	**C**	**C**	**G**	**C**	**C**	**C**	**A**
MNG 3143	3.ANT?					T	C	T	T	G	T	C	A	**C**	**C**	**G**	**C**	**C**	**C**	**A**
MNG 2881	3.ANT?					T	C	T	T	G	T	C	A	**C**	**C**	**G**	**C**	**C**	**C**	**A**
MNG 3052	2. ANT3					T	C	T	T	G	T	C	A	**C**	**C**	**G**	**C**	T	T	**A**
MNG 3020	2. ANT3					T	C	T	T	G	T	C	A	**C**	**C**	**G**	**C**	T	T	**A**
MNG 3013	2. ANT3					T	C	T	T	G	T	C	A	**C**	**C**	**G**	**C**	T	T	**A**

**Figure 7:**
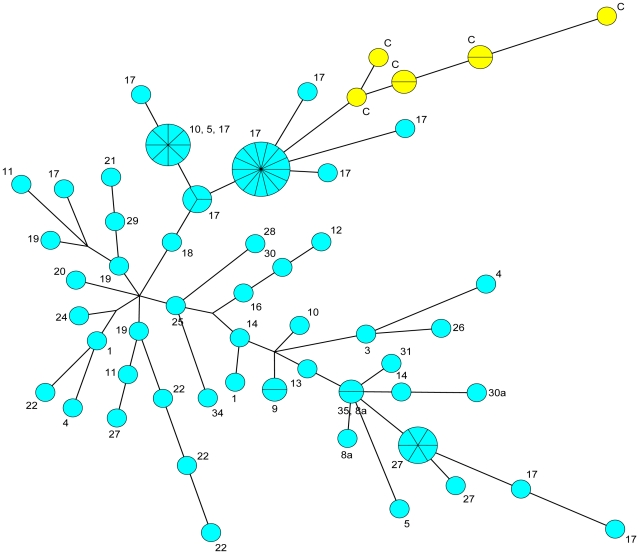
MST tree of the main Mongolian clade. MST tree of the 78 Mongolian *Y. pestis* strains, determined as 3.ANT genotype and showing CRISPR spacer b48 (blue). The seven strains previously described by Li et al. and associated to the Mongolian cluster were included (yellow) [5], suggesting distinct clustering. Numbers refer to the sampling sites given in Fig. 4.

The three other clusters (87 strains) do not contain a linking strain which would allow a robust SNP branch assignment. One cluster corresponds to the Ulegeica *microtus* biovar (four isolates and MLVA25 genotypes) not included in Morelli et al. [6] (Figure 5/green box). This assignment is deduced from the co-clustering with three Ulegeica strains investigated by Li et al. [5]. All three previously investigated Ulegeica strains originate from Mongolia. Strains of the two other MLVA-clusters correspond to *Y. pestis* subspecies *pestis* isolates (Figure 5 and 6/brown and turquoise boxes). The smaller cluster comprises five *Y. pestis pestis* Antiqua isolates (4 MLVA25 genotypes) from foci 6, 8a, 30, 32. They are most closely related to isolates from Tuva focus #37 in Russia (Figure 4 and 5/brown) immediately adjacent to the western border of Mongolia [4], [5]. The third unassigned cluster is by far the most numerous and frequent in Mongolia (78 isolates, 41 MLVA25 genotypes; Figure 6/turquoise). It is closest to a small group of seven isolates shown in supplementary Figure 2 in Li et al. [5]. These seven isolates (5 MLVA25 genotypes), C1976008, C1976001, C1989002, C1961006, C1972002, C1972001, C1972003 (Figure 6), were collected in Akesai, Gansu province and Wulan, Qinghai province, China [5]. MLVA25 clustering tends to link these two clusters to 3.ANTa or 0.ANT branches. The relative MLVA genotype diversity of the two groups is shown in Figure 7. The diversity observed in Mongolia is much larger than the diversity in China, but this may be due to the larger number of available Mongolian strains. The two groups are clearly resolved by MLVA suggesting low level of strain circulation and cross contamination between the Mongolian and Chinese foci.

### SNP typing of selected strains

The assignment of three clusters to the 0.PE1, 0.PE4 and 2.ANT3 SNP-defined branches as suggested by MLVA25 clustering with linking strains could be confirmed for selected strains by typing relevant SNPs (Table 2). The remaining three MLVA clusters were positioned on the SNP tree by typing a few selected strains from each cluster for key SNPs according to Morelli et al. [6] (Table 2).

Ulegeica strain MNG 2972 representative of the Ulegeica cluster could be assigned to branch III–VI by analyzing all 56 SNPs in this branch [6]. Eleven SNPs, s85, s90, s463, s846, s849, s940, s951, s1099, s1221, s1248, and s1351 showed the derived genotype, the other 45 showed the ancestral state. This enables the precise positioning of the Ulegeica branching node in between III–VI and indicates that Ulegeica is the most recent *microtus* branch characterized so far. We propose to call the Ulegeica clade *0.PE8* in agreement with the published SNP branch nomenclature (the Hissarica biovar could be assigned as *0.PE9*, if additional SNP analyses will confirm this assumption) (Table 1).

Eight selected strains from the remaining two MLVA25 clusters, MNG 649, MNG 3054, MNG 2986, MNG 2853, MNG 2645, MNG 3088, MNG 3143, MNG 2881, revealed a derived genotype for the tested SNPs for all branches connecting nodes 0.PE3a and 3.ANTa, and an ancestral genotype for the tested SNP for the branches XI-3.ANTa (as well as XIII–XI, 1.IN2a-XIII, XII–XI) and VIII-3.ANTa (as well as VIII-2.ANT3a and 2.ANT3a-2.ANT2a). This demonstrates that the two clusters are branching out within the 0.ANT3a, VIII and XI nodes (Table 2). Further SNP typing and whole genome draft sequencing of a few selected strains will allow to determine the exact positioning of the two Mongolian clades.

### CRISPR analysis

The three CRISPR loci YPa, YPb, YPc could be amplified, and completely sequenced in 96 of the 100 isolates [18]. Four DNA samples yielded double bands in YPa (MNG 1683, MNG 1691), or YPb (MNG 3050, MNG 3054). CRISPR analysis revealed 14 different genotypes, six of which have not been described so far. Seven new spacers for loci YPa, and YPb were observed [18], [26] (Figure 5 and 6, Table 3, 4,5, Table S1). The a6′ spacer is a variant of the a6 spacer [26]. Protospacers were identified for all spacers either on the *Y. pestis* chromosome (a6′, a85–86, a88, b48–49), or on the pCD1 (a87) plasmid. They code for conserved hypothetical proteins, putative phage proteins, or, interestingly, CRISPR-associated helicase Cas3 (the protospacers position in the CO92 genome is indicated in Table 5). The previously published *Y. pestis* CRISPR genotype 45 from Bayanölgie province, Mongolia (Figure 4/focus BP) [18], is identical to genotype 8-4-3, MNG 3096 (Figure 4/sampling site 8, Table S1). The previously published CRISPR genotype 46, from the Russian Mountain-Altai-focus-36 (Figure 4/#36) presents a nearly identical CRISPR profile (3-5-2) to strains MNG 3125, MNG 3126, MNG 2197, and MNG 2198 (Figure 4/sampling sites 7, 8a; Table S1). YPa from four Mongolian strains include the previously published a80 spacer sequence [18]. The *microtus* Ulegeica strain MNG 2972 showed the unique YPa profile a1.a2.a3.a4.a5.a37′.a82.a85.a86 (Figure 5, Table 3), including the two new spacers a85 and a86 (Table S2). Two additional spacers a87 and a88 were found in other Ulegeica strains, (Table 3, 4, 5, S1, and S2). New spacers, b48, and b49 are associated with Ypb (Table 3, 4,5, S1, and S2). Spacer b48 is present in 75 out of 100 strains, which also form a highly homogenous MLVA cluster (Figure 6/turquoise, Table S1). For the YPc locus, the least diverse CRISPR locus, no new alleles were observed compared to previously published data (Table 3) [18].

**Table 3:** CRISPR spacer signatures.

Ypa	code	Ypb	code	Ypc	code
a1.a2.a3.a4.a5.a6.a7.	1	**b1.b2.b3.b4.b48.**	**1**	c1.c2.c3.	1
a1.a2.a3.	2	b1.b2.	2	c1.c2.	2
**a1.a4.a6′.** *	**3**	b1.b2.b3.b4	3	c1.c3.	3
a1.a2.a3.a4.a5.	4	b1.b2.b3.b4.b10.	4		
**a1.a2.a3.a4.a5.a6.a7.a88.**	**5**	b1.b3.b4.b10.	5		
**a1.a2.a3.a4.a5.a6.a7.a87.**	**6**	b1.b2.b3.b4.b47.	6		
a1.a2.a3.a4.a5.a6.a7.a80.	7	**b1.b2.b3.b4.b48.b49.**	**7**		
a1.a2.a3.a4.a5.a37′.a82.	8				
**a1.a2.a3.a4.a5.a37′.a82.a85.a86.**	**9**				
a1.a2.a6.a7.	10				

*bold print: first described in this study.

**Table 4:** CRISPR genotypes.

CRISPR genotypes		
This work	Source	Usually associated with
1-1-1	This study, similar to genotype 22 [16]	Mongolian cluster
4-1-1	This study	Mongolian cluster
5-1-2	This study	Mongolian cluster
6-1-1	This study	Mongolian cluster
10-7-1	This study	Mongolian cluster
2-2-1	Genotype 1 [16]	Antiqua focus H
3-4-2	Similar to genotype 37 [16]	Xilingolensis
3-5-2	Similar to genotype 46 [16]	Altaica
7-3-1	Previously described spacers [16]	Antiqua China focus ??
7-6-1	Previously described spacers [16]	Antiqua China focus ??
4-3-3	Previously described spacers [23]	Ulegeica
8-3-3	Similar to genotype 45 [16]	Ulegeica
8-4-3	Genotype 45, Mongolian strains [16]	Ulegeica
9-4-3	This study	Ulegeica

**Table 5:** Protospacers for newly identified spacers a6′, a85–88, and b48–49.

Spacer	Sequence	Corresponding gene	Gene products
a6′	TCGGTCAAACAAATTTAGGCGACGATTTAA	YPO2469	YP conserved hypothetical protein
a85	CCCCTGCCTTTTGCAGCCAGTCGCGCCACTCT	YPO2106	putative phage protein (pseudogene)
a86	AGCCCGCCCCGCACGATAAGCATTGAACAACG	YPO2467	CRISPR-associated helicase Cas3
a87	CACTTGTTGATGTGACTCTGACAAATGGGATAA	pCD1	Yersinia outer protein
a88	TGAAGGTATGGAATCTTGTGACCAATGGGTTT	YPO2108	hypothetical phage protein
b48	TCGCGCCAGTATGGATGGACAAGTTCCAGCGGG	YPO2108	hypothetical phage protein
b49	TGGCTTTATTGTGGTCAGCTTTGTCGTATCCGG	YPO2112	YP conserved hypothetical protein

Altogether the clustering of CRISPR genotypes is highly congruent with MLVA clustering (Figure 5 and 6).

### Progressive hierarchical resolving assays using nucleic acids (PHRANA)

In this study, MLVA25 analysis allows a classification of the 100 Mongolian strains into 65 genotypes defining six clusters, corresponding or closely related to known biovars, or recently described lineages (Table 1, 2). Partial SNP typing was then applied to selected strains in order to anchor more precisely clusters devoid of linking strains. This approach is a PHRANA approach in which MLVA, rather than SNP typing as initially proposed, is used as a first line assay. The CRISPR typing yields 14 different genotypes. The CRISPRs alleles in *Y. pestis pestis* are exceptional by the fact that most spacers (except for the oldest a1–a6) originate from the chromosome (or the plasmids) as compared for instance to *Y. pseudotuberculosis* spacers [26]. The new spacers identified in this work also originate from the chromosome. Very interestingly, Ulegeica is unique among the *microtus* lineages in that its recent spacers a82-a85-a86 originate from the chromosome. In this respect, Ulegeica is close to *Y. pestis pestis*.

### Conclusions

The present investigation illustrates and confirms the large variety of the Mongolian *microtus* biovars Ulegeica, Altaica, and Xilingolensis present in a close proximity (Figure 5, 6, and 7). It suggests that western Mongolian foci or the adjacent Siberian foci are likely places of emergence of Ulegeica, the most recent clade. Xilingolensis would have spread throughout Mongolia, to focus L in Manchuria (Figure 4). Qinghaiensis is found further south in central China (focus M) [5]. The Mongolian *microtus* Ulegeica clade is shown to be the most recent *microtus* branch along the linear tree leading from *Y. pseudotuberculosis* to *Y. pestis* subspecies *pestis* biovars Intermedium, Antiqua, Orientalis, Medievalis. Ulegeica contributes to the filling of a large gap.

The absence in Mongolia of *Y. pestis pestis* lineages branching along the III-0.ANT3a segment is consequently surprising and might indicate that the presence of *Y. pestis pestis* in Mongolia is the result of a secondary introduction of strains from China [6] perhaps in the last hundred years as human infections are reported since 1897 in this country. The Gansu province south of Mongolia, in which closest neighbors from the most frequent Mongolian *Y. pestis pestis* are present, is a likely source. Alternatively, Mongolian 0.ANT representatives might have been replaced by the more recently emerged 3.ANT lineage, and become extinct or at least very rare in Mongolia. More detailed whole genome sequencing and SNP analysis will be required in order to test these two hypotheses and precisely deduce the direction of the dissemination of the 3.ANT lineage across Mongolia and China. In addition, a systematic MLVA typing of Y. *pestis* strain collections may enable the identification of other rare clades as previously illustrated [5], [6].

## Materials and Methods

### Strains and DNA

In this study only strains isolated from wildlife animals or their parasites were investigated to focus on genotypes occurring in nature and with a clear geographic assignment, in contrast to strains recovered from patients who may have travelled recently (Table S1). The investigated plague-strains were conserved in glycerol stocks. For this work, they were recovered on Hottinger's agar at 28°C for 24 h and subcultured on Columbia blood agar at 28°C for 24 h. Thermolysates were prepared by heating a bacterial suspension for 30 min at 95°C. The 100 *Y. pestis* strains investigated in this study were collected between 1960 and 2007 from 37 sampling sites in Mongolia (Figure 4) distributed over 13 aimags (provinces). They were isolated from various parasitic plague-vectors, such as *Oropsylla silantiewi*, but also from lice or ticks (Table S1). Parasites were collected from mammalian host species, such as *Marmota sibirica*. All strains revealed both *Y. pestis* specific virulence plasmids pMT1, and pPCP1 when investigated by previously published real-time PCR [27].

### MLVA markers and PCR amplification

Twenty-five VNTR markers were applied [5]. Three loci were co-amplified in a single multiplex PCR and the resulting products were analysed on a CEQ8000 capillary electrophoresis machine (Beckman-Coulter, Marseille, France) essentially as described [14], [28]. The resulting data were analyzed and merged with the previously generated MLVA database including more than 500 *Y. pestis* strains the majority of which from Central Asia using BioNumerics software package version 6.5 (Applied-Maths, Sint-Martens-Latem, Belgium) [5]. The tree was rooted using two *Y. pseudotuberculosis* isolates as an outgroup. MLVA data corresponding to pestoides strains A, B, C and D in Achtman et al. [7] were kindly provided by Dr. Paul Keim.

### SNP typing

At least one SNP was selected for each relevant branch to determine branching of the Mongolian plague strains within the previously published SNP minimum spanning tree [6]. Each SNP was amplified by conventional PCR, sequenced and analyzed by alignment with *Y. pestis* type strain CO92 (Table 2).

### CRISPR analysis

The three CRISPR loci YPa, YPb, and YPc were amplified for each plague-strain by conventional PCR as described previously [18], [26], [29]. The sequences were analysed with the software CRISPRcompar and CRISPRtionary via the CRISPR website http://crispr.u-psud.fr/ [30]–[33]. The previously published CRISPR data from *Y. pestis* strains was used as reference (“spacers dictionary” Table S2, this report and Table S1 in Cui et al. [18]). Newly found spacers received the next consecutive number. The CRISPR genotype was presented in a three digit code e.g. 1-1-1 (lack of amplification for one locus was coded X as in 1-X-1) (Table 4, Table S1).

### Ethics Statement

The bacterial strains in this study were obtained from non-vertebrate vectors, collected from various mammal-species (Table S1). Mammals were trapped in one-door live traps, as previously described [34]. The protocols for trapping animals and isolation of strains were authorized by the *Mongolian Ministry of Health Ethical Committee* (record no. 223/2007) and follow international guidelines and requirements, as stated in “iagnostics, Treatment, and Surveillance of Plague”(record no. MNS5348-41/2010, 8.2.1.2) for the investigation regarding notifiable diseases. Investigation of dead animals was authorized by the order of the Mongolian Minister of Health and the department of standards and measurements (record no. 151/2008; item 5.2.4: “collection of samples with epidemiological risk for laboratory investigation”).

## Supporting Information

Table S1 **Properties of** ***Y. pestis*** **strains used in this study.**
(XLS)

Table S2 **CRISPR_Dictionary updated from Cui et al.** [**18**] **including Mongolian strains data.**
(XLS)
